# Experiences and needs of Saudi mothers when a child or adolescent is diagnosed with type 1 diabetes mellitus: a qualitative study

**DOI:** 10.1080/17482631.2022.2107151

**Published:** 2022-08-04

**Authors:** Mariam Asaad, Rita Forde, Abdullah AlFares, Bassam Bin Abbas, Jackie Sturt

**Affiliations:** a Florence Nightingale Faculty of Nursing, Midwifery and Palliative Care, King’s College London, London, UK; b Department of Pediatrics, Security Forces Hospital, Riyadh, Kingdom of Saudi Arabia

**Keywords:** Children and adolescents, mothers’ perspectives, qualitative research, Saudi Arabia, type 1 diabetes mellitus

## Abstract

**Aim:**

To explore the experiences of Saudi mothers with children or adolescents who have Type 1 diabetes mellitus at time of diagnosis.

**Background:**

The Kingdom of Saudi Arabia (KSA) has one of the highest incidence rates of Type 1 diabetes mellitus in children and adolescents in the world. Few studies have considered the most appropriate methods of support for parents in the KSA and none report the experiences of Saudi mothers.

**Design:**

Phenomenological inquiry.

**Method:**

Qualitative interviews were conducted with 11 Saudi mothers and data were analysed following Giorgi’s 5-step method.

**Results:**

The lived experiences of Saudi mothers coalesced around three overarching themes and eight subthemes: 1. In the dark (mother’s instinct, challenges of diagnosis phase, cultural reflections); 2. Empowerment (methods of support, mother’s health and wellbeing); 3. Coping and acceptance (stigma and cultural perceptions, coping strategies, transformation and adaptation).

**Conclusions:**

Effective interventions delivered in other countries to support mothers may be effective in the KSA. However, the central role that Saudi mothers play in the management of their child’s condition, and the place of Islam in Saudi society, indicate the need for customized methods of support that take into account psychosociocultural needs of both mother and child.

## 1. Introduction

The incidence of Type 1 diabetes mellitus (T1DM) is increasing worldwide with more than 60% of cases residing in Asia (IDF, 2021). Among children and adolescents, the annual global increase is estimated at around 3%, although there are significant geographical variations (IDF, 2021). Whilst incidence is greatest in northern European countries including Finland and Sweden, the Kingdom of Saudi Arabia (KSA) has the eighth highest incidence rate (IDF, 2021). T1DM is a chronic autoimmune condition where the immune system attacks the insulin producing beta cells in the pancreas resulting in the body producing very little or no insulin (Chiang et al., 2018; IDF 2021).Estimates for the annual increase in the KSA are as high as 16.8% (Abduljabbar et al., 2010), although a lack of available research means that the incidence may be under-estimated (IDF, 2021).

Reasons for increasing incidence are similar to those elsewhere in the world including changes to diet and breastfeeding practices, and exposure to environmental pollutants and toxins (Robert et al., 2018). The practice of consanguinity in the KSA has also resulted in an increased gene pool for susceptibility to T1DM (Zayed, 2016). Consequences of the rapid rise in cases are particularly concerning. Modifiable risk factors have been reported by (A. E. Al-Agha et al., 2015) in a study of 228 children in Jeddah over a one-year period. A total of 41.6% experienced sub-optimal glycaemic control and 65.4% had at least one episode of diabetic ketoacidosis. Long-term complications were also detected including retinopathy (4.4%) and microalbuminuria (16.2%). A more recent review of the literature indicates that these complications continue to persist (Robert et al., 2018). Although the Ministry of Health is responsible in the KSA for diabetes treatment and education, these results indicate that much still needs to be accomplished including the support of parents to better manage the condition.

Saudi culture is deeply rooted in religious values and traditions, which underpin people’s reactions to bad news and their coping strategies. Cultural norms determine that mothers are the primary carers who will often seek support from each other. However, no qualitative research has been conducted into the experiences of Saudi mothers who have a child with T1DM. Evidence from the wider Islamic world points to the struggle that mothers experience and associated attempts to determine the cause of their child’s T1DM (Khandan et al., 2018). Some mothers attribute causation to the “evil eye”, a common belief in Islam that individuals have the power to look at people and cause them harm (Haugvik et al., 2017). Financial difficulties and a lack of universal health services can also impact on a family’s ability to provide adequate care, to the extent that some children suffer complications including seizures and coma (Haugvik et al., 2017).

The shock that parents experience when a child is diagnosed with T1DM is well documented in Western literature (Whittemore et al., 2012). The life-threatening character of the condition is experienced as an immense burden, and the psychological aspect of diabetes distress on parents can be life-altering (Iversen et al., 2018). However, whilst similarities are likely to exist between this evidence and the experiences of mothers in the KSA, key differences mediated by culture are also anticipated. The rise in T1DM among children and adolescents in the KSA has been described as a soaring epidemic, and the complications that arise are a significant public health concern (Robert et al., 2018). An understanding of the experiences of Saudi mothers at the time of diagnosis, including their needs and coping strategies is necessary to develop customized methods of support.

## 2. Methods

### 2.1 Aim

The aim of this study was to explore the experiences of Saudi mothers whose children have T1DM, and to deepen understanding of their coping strategies and needs at the time of diagnosis.

### 2.2 Design

The study was designed as a descriptive phenomenological inquiry to explore the “lived experience” of participants and capture the meaning they attributed to the phenomena of interest (Giorgi, 2005). Phenomenology is the philosophical study of the “experience” (Harper et al., 2011). This epistemological position was informed by the lead researcher’s personal experience, whose daughter and son had both been diagnosed with T1DM. Alternative designs such as interpretive phenomenologies attempt to interpret the experiences of participants, potentially making them more meaningful whilst recognizing the researcher’s inability to gain an “insider’s perspective” (Conrad & Barker, 2010; Harper et al., 2011). The descriptive phenomenological approach was chosen to provide a more-true, less interpreted portrayal of the participants’ experiences and took into account the stance of the lead researcher.

It is reported in accordance with the Consolidated Criteria for Reporting Qualitative Studies (Tong et al., 2007).

### 2.3 Participants

Participant inclusion criteria were: Saudi mothers who held a national Saudi identification card and had a child with T1DM between the ages of 5 and 18 years; and, the child had been diagnosed for at least six months with no other coexisting long-term conditions. Participant recruitment began at the endocrinology clinic of the hospital in Riyadh, KSA. Potential participants were approached by the lead researcher and those who expressed an interest were provided with a participant information sheet and invited to provide consent for a follow-up telephone call to discuss their inclusion. Study information was also shared through Twitter and WhatsApp groups for mothers whose child(ren) had T1DM. Participant information was shared with mothers who expressed an interest and phone numbers were exchanged to explore recruitment. A purposive sample of 11 participants was recruited through these methods.

The sample size was determined using the guidelines of the “information power” theory by (Malterud et al., 2016). This is based on five dimensions that impact information power, (1) The study aim; the narrower the study aim, the smaller the sample; (2) sample specificity; constraining inclusion criteria require a smaller sample; (3) established theory; limited theoretical perspectives require larger samples than specific theories; (4) “quality of dialogue” strong and clear communication between researcher and participant requires fewer participants than ambiguous and unfocused dialogues; (5) analysis strategy, such as exploratory cross-case analysis will require a larger sample than an in-depth analysis approach. Therefore, as this study had narrow and specific aims, strict inclusion criteria, and in-depth qualitative analysis approach, a smaller sample size was indicated.

### 2.4 Data collection

A semi-structured interview guide was developed which was refined through peer review with three members of the research team. In addition, it was piloted face-to-face with a mother of a child with T1DM. To address the study aim, the interview guide included 17 questions and probes to encourage participants to share experiences related to three main topics: the time of diagnosis, coping strategies and needs (Table I). The interviews were conducted in Arabic either face-to-face (n = 4) or by telephone (n = 7), in a private place of convenience to the participant. Thus, interviews were conducted on a one-to-one basis in October 2019 either in the lead researcher’s home or the participant’s home as per their convenience and preference to ensure privacy, and were audio digitally recorded. The lead researcher conducted all interviews which lasted between 25 and 45 minutes. Reflections and researcher notes were documented in a reflexive diary and journal after each interview.

**Table I.** Interview guide.

Time of diagnosis
Could you tell me about when your child was diagnosed with T1DM? How did you discover your child had T1DM? Could you tell me about the events that happened on that day. Please tell me about the discharge experience (if the child was hospitalized). What was provided upon discharge (brochures, leaflets, contact numbers, website link)? What support was provided?
**Coping strategies** How did you cope in the first few weeks after the diagnosis? What helped you cope? What did you find most difficult during this time? What support did you have?
**Needs** Could you reflect on what you needed at the time of diagnosis? What might have been helpful for you at the time of diagnosis? What about a leaflet with tips from other mother’s experiences, would that have been helpful? What about a video with shared experiences, would that have been helpful? What about a face-to-face support group with other mothers, would that have been helpful? In your opinion what would have been most helpful? What words of advice would you give to a mother going through the same experience now, and why?

### 2.5 Ethical considerations

Ethical approval was obtained from the Internal Review Board (IRB) at Security Forces Hospital on 21 February 2019 (H-01-R-069) and from the ethics committee at King’s College London on 28 March 2019 (HR-18/19-9201).

All participants provided informed consent and were made aware of their right to withdraw from the study without it affecting the care they or their children received. Transcripts were anonymized with participants allocated identification numbers.

This study may have been considered a potentially sensitive topic for some participants and may have elicited an emotional response such as sadness or distress. The framework for sensitive interviews developed by (Dempsey et al., 2016), was used as a guide if necessary. The role of being a researcher and a peer during the interviews enhanced the reciprocal nature of the interview and helped create a less intimidating, supportive environment (Elmir et al., 2011). With regards to qualitative research, (Gray, 2018) states “informed consent is a fluid process requiring constant monitoring” (p189). That holds true to this research, where flexibility and willingness to take a break were implemented during the interviews when necessary. In this study there was no need to end an interview, however when a participant became emotional, tissues and water were provided and they were asked if they needed to take a break, in one case the interview was paused for a few moments while the participant regained composure, nobody expressed a need for support from the diabetes educator or a psychologist, instead the researcher debriefed with participants after the interview to ensure closure.

### 2.6 Data analysis

The interviews were transcribed verbatim by the lead researcher in Arabic and compared with voice recordings for accuracy. The transcripts were then annotated with emotional responses such as crying and laughing, and when there were moments of silence. Transcripts were translated from Arabic into English by the lead researcher and these transcripts were reviewed by two members of the research team (AA, BB) for sense checking.

The lead researcher performed the analysis following Giorgi’s 5-step method (Giorgi, 2005). This began by becoming familiar with the data through reading and re-reading the transcripts and followed a process of reduction by dividing participant descriptions into units of meaning, which were gradually reduced further by combining related units and eliminating duplicates before transforming the units to describe the phenomena of interest. Analysis was conducted using the data management software NVivo 12 with a codebook developed to assist the process. The analysis was inductive and conducted iteratively until no new codes and themes were identified. There is an uncertain logic underpinning the complexities of the concept of saturation (Saunders et al., 2018) or reaching information redundancy, that is when no additional data are being found (Glaser & Strauss, 1967) and no new codes or themes are identified (Birks & Mills, 2015). This study drew on the “information power” model by (Malterud et al., 2016) which indicated that a smaller sample size would suffice based on the study aims and design.

### 2.7 Scientific rigour

Two co-researchers (JS, RF) reviewed the analysis codebook and independently coded four transcripts to provide additional perspectives, discuss differences in data interpretation, ensure data saturation and promote inter-rater reliability. The validity of the research was assured by following the recommendations of (Whittemore et al., 2001) performing a literature review, verbatim transcription of interviews, bracketing, reflexive journaling, the use of computer programmes, expert checking as described above, providing evidence to support interpretations and acknowledging the researcher’s perspective.

Attention was paid to Lincoln and Guba’s four criteria for assuring qualitative research rigour: credibility, dependability, confirmability and transferability (Lincoln & Guba, 1985).

#### 2.7.1 Credibility

Analytical debriefing with the research team to attenuate personal biases, which also provided opportunities to discuss and defend emergent interpretations of the data.

#### 2.7.2 Dependability

Preliminary interpretations were discussed among the research team in detail and in relation to the original data.

#### 2.7.3 Confirmability

The study findings are supported with extracts of the participants’ contributions, and an analysis codebook was developed, and an audit trail maintained to support analytical decision making.

#### 2.7.4 Transferability

This study used purposive sampling and a detailed account of the participants’ characteristics has been provided to contextualize their contributions.

## 3. Results

The mothers’ and their children’s characteristics are presented in Table II. The mean age of the mothers was 39 years (range 27–49) and the mean age of their children at time of diagnosis was 10 years (range 5–18). Participants were from all regions of the country and from diverse socio-economic backgrounds.

**Table II.** Characteristics of the mothers and their children.

Participant number	Age (years)	City of residence and region	Gender of child	Age of child at diagnosis (years)	Duration of diabetes	HbA1c at diagnosis and DKA status	No. of children with T1DM	Recruitment method
P1	29	Riyadh (Central)	Female	10	6 months	140 mM/M (15%) DKA	2	WhatsApp
P2	32	Riyadh (Central)	Female	11	1 year	130 mM/M (14%)	1	SFH
P3	49	Riyadh (Central)	Female	18	2 years	140 mM/M (15%)	1	SFH
P4	48	Riyadh (Central)	Male	12	1 year	75 mM/M (9%)	1	SFH
P5	46	Abha (Southern)	Female	10	1 year	96 mM/M (10.9%)	1	Twitter
P6	27	Madinah (Western)	Male	7	1.5 years	75 mM/M (9%)	1	Twitter
P7	46	AlAsha (Eastern)	Male	6	7 months	75 mM/M (9%)	1	Twitter
P8	32	Riyadh (Central)	Female	5	8 years	130 mM/M (14%)	2	WhatsApp
P9	48	Riyadh (Central)	Female	14	2 years	68 mM/M (8.4%)	3	SFH
P10	37	Riyadh (Central)	Female	5	1 year	130 mM/M (14%) DKA	2	WhatsApp
P11	38	Jeddah (Western)	Female	9	6 years	75 mM/M (9%)	1	WhatsApp

DKA: diabetic ketoacidosis HbA1c: glycated haemoglobin mM/M: Millimole per mole SFH: Security Forces Hospital T1DM: Type 1 diabetes mellitus

**Figure 1.**
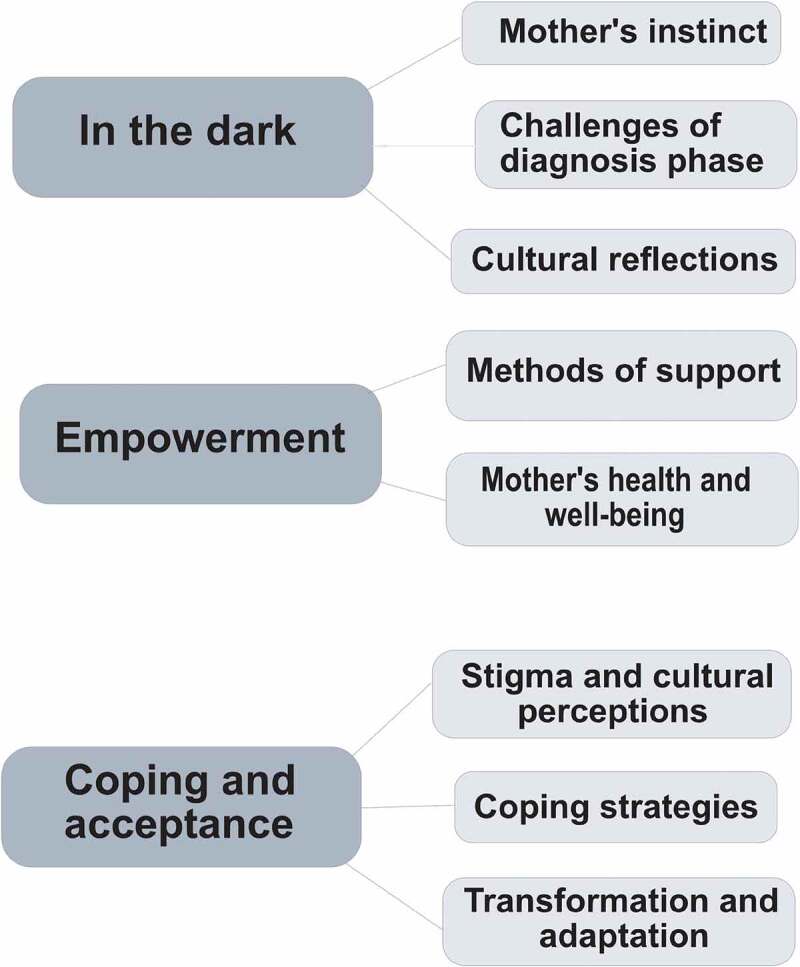
Themes and sub-themes of mothers’ experiences.

Three main themes emerged from the data: in the dark, empowerment, and coping and acceptance. Figure 1 presents each theme together with their constituent sub-themes.

### 3.1 In the dark

The time of diagnosis was a period of darkness for all participants as they dealt with the shock of their child’s diagnosis and entered a realm of the unknown. The cultural context underpinning darkness stems from ancient cultures in the Arabian Peninsula, where darkness meant ignorance and a lack of knowledge in addition it indicated a lack of clarity, despair, sorrow and sadness, when Islam came to this region it transformed the culture by bringing light through knowledge, clarity and hope to its people. Thus, the darkness theme reflected both the participants’ sad, desperate emotional state and their lack of knowledge of diabetes at the time of their child’s diagnosis.

#### 3.1.1 Mother’s instinct

A mother’s gut feeling that something was wrong with her child was expressed by many of the participants. They observed strange symptoms such as increased thirst and urination. One participant described how her son’s school allowance was no longer enough to meet his water needs (in the KSA, bottled water is the only method of obtaining drinking water):

‘I knew there was something strange. When he came back from school, he would say that five or six or seven Riyals was not enough for him to buy water at school, he said: ‘I need a bigger allowance, three or four bottles of water are not enough’. (P4)

Four mothers had more than one child with T1DM and their gut feeling that it might happen to another child was keenly felt. One participant shared her concern with her health care professional (HCP), only to be faced with a response which she felt ridiculed and undermined her concern:

‘I asked the doctor to do a blood test to check my other children for T1DM. The doctor’s response was: ‘why do the test? Unless you want to become like the mice in trials’. When my third child was sick, I broke down because I’d asked everybody’. (P1)

#### 3.1.2 Challenges of diagnosis phase

The day of their child’s diagnosis was recalled by all participants no matter how long ago. It evoked emotions as they recalled their feelings which included shock and disbelief. Reactions appeared particularly acute for mothers who had a second or third child diagnosed with T1DM because they knew what the child would face. The experience was often compounded by other family health problems that provided a bewildering context within which to accommodate the diagnosis. A lack of emotional support and empathy when the diagnosis was shared by an HCP were described by some mothers, which made the impact of the condition challenging to comprehend. Lacking knowledge, some mothers hoped their child’s condition was temporary and could be resolved with treatment.

‘To the extent that I thought if they gave her insulin and her blood sugar went back down to 80, she would be fine, that was it. I had no idea that this was a lifelong disease’. (P8)

#### 3.1.3 Cultural reflections

The male is the head of the household in the KSA and considered the major decision-maker for all family related decisions. Some participants had experienced denial from their husbands about their child’s symptoms and a reluctance to take them to the hospital:

‘I spoke to my husband and told him that I spoke to my relative and she listed all the symptoms and my son has the same symptoms. He said: ‘I have enough on my plate, and I am sick receiving treatment. Leave him for now it’s just your imagination’. (P4)

Participants reflected on what may have caused their child’s condition and some attributed it to the “evil eye”:

‘You take pictures of your daughters and post them on social media, you don’t say the special prayers (for envy) … I agree with all this, and I suddenly felt it might be the evil eye.’ (P1)

### 3.2 Empowerment

When participants overcame the initial shock of their child’s diagnosis, they began to seek knowledge and support. They chose to empower themselves with knowledge to better understand their child’s condition and how to manage it. In addition, having a strong knowledge base allowed them to address sociocultural misconceptions about diabetes with evidence-based knowledge. In the face of social stigma, many mothers found that through dialogue and by being open and transparent they could gradually change social stigmatization against their child which mostly occurred in schools and after school activities.

#### 3.2.1 Methods of support

Due to a lack of official support mechanisms, many mothers turned to peers through the use of social media. The most helpful platforms were Twitter, Snapchat, YouTube and WhatsApp groups. One participant described the pivotal role peers on a WhatsApp group had played, preventing her from harming her son, due to her lack of understanding:

‘T1DM is very easy, if it is high or if he eats, I give him a lot of insulin, if it is low I give him a little insulin and juice. The boy almost died! I almost killed him! But God bless them, there were three girls on the group who were very, very supportive.’ (P6)

HCP input was essential for participants, however not all reported the same level of support. Some mothers praised their diabetes educator whilst others reported no educator at their hospital and felt that a lack of adequate training for the HCP team left them feeling unprepared to manage their child’s condition. Some participants also described negative experiences with HCPs:

‘I came away from meeting them feeling upset and frustrated. They were not supportive.’ (P11)

Family emerged as a key method of support for many of the mothers. One participant mentioned how a cousin reached out from across the country to support her, another acknowledged the importance of her husband ‘s support when her child was first diagnosed:

‘But I was lucky because my husband was supportive. It was me and him together supporting each other.’ (P10)

A child’s return to school was an especially difficult time for mothers who had to let them go for the first time since diagnosis. Some shared a positive experience of school support, and how coincidentally they encountered teachers or supervisors who had a child with T1DM and knew exactly how to manage their child’s condition. On the other hand, a few experienced a lack of availability of school nurses to support them, and one mother recalls how they had to move her daughter to a new school:

‘Her old school didn’t have a nurse or any other students with T1DM, she would suffer hypos and nobody knew what to do. We suffered terribly’. (P5)

In a society that uses faith for physical and spiritual wellbeing, psychological support is not usually sought. It would be considered a sign of weakness if one resorted to psychological help. However, a few mothers expressed a need for such support, and some recognized its importance for their child as well. One mother reflected on the unavailability of emotional support:

‘But they lack the ability to address the emotional side of the illness for the mother and the child, nothing, there was nothing!’ (P5)

#### 3.2.2 Mothers’ health and wellbeing

Participants faced a wide variety of challenges. Some involved technical and cognitive skills such as learning how to inject, managing hypo/hyperglycaemia and learning how to carbohydrate (carb) count. Other challenges had a physical and emotional impact such as lack of sleep and the worry of monitoring a child after an episode of hypo/hyperglycaemia, which mainly occurred at night. Sleep deprivation was experienced by most mothers around the time of diagnosis. An especially challenging sleep scenario was described by a mother who had also had a baby at the time of her daughter’s diagnosis:

I had just had a baby at the time of her diagnosis, and I would wake up every 30 minutes to check, it was exhausting. It is my nature … the hardest thing in the beginning is the sleep, nobody tells you this. (P2)

Mothers undertook most of the care and many had to find the strength to overcome obstacles on their own. Giving injections was very difficult, especially for those with younger children. One participant recalled the heartache and pain she felt when injecting her daughter:

‘Honestly, training on giving the injections, it was heart-breaking, devastating. It was the most difficult thing’. (P10)

Keeping blood glucose levels within range was both a physical and mental difficulty experienced on a daily basis. Some found that carb counting helped them keep blood sugar levels within range, and others found that keeping track of readings helped. One mother (P3) described her overwhelming fear of hypoglycaemia as a *“hypo phobia”*. With a child’s diagnosis came new habits and norms that dominated the mothers’ lives.

Knowledge was an important factor for promoting a mother’s wellbeing. Once they had overcome the initial shock and began to seek support, they empowered themselves through knowledge. Knowing what T1DM was, learning how to deal with insulin dosing and becoming more comfortable with managing hypo and hyperglycaemia incidents helped them attain a better emotional state. Part of the participants’ path to knowledge was taking the initiative to learn about technologies related to T1DM to help ease the burden of care, such as continuous glucose monitoring (CGM) and insulin pumps.

### 3.3 Coping and acceptance

All of the Participants’ experiences through a journey of empowerment translated into a new phase of coping and acceptance. Many described the coping strategies they used, and their role in helping them find an inner strength to help their transition to the phase of acceptance. For many of the participants their coping strategies were underpinned by sociocultural constructs from within the Saudi culture.

#### 3.3.1 Stigma and cultural perceptions

Knowledge and empowerment enabled some mothers to tackle social stigma and misinformation. One recalled that when her daughter returned to school, her classmates were afraid of her injections and worried that she had a contagious condition. The participant spoke to the other children’s mothers to ease their misunderstandings and help her classmates’ acceptance. Another daughter faced differential treatment by grandparents who prevented her from eating desserts. Her mother was able to change their misconceptions by educating them about T1DM and insulin.

#### 3.3.2 Coping strategies

Difficulties identifying effective coping strategies were reported by all participants. However, these varied as mothers’ experienced different feelings, needs and emotions. Coping strategies ranged from prayer, faith, optimism and hope from seeing others with T1DM. Faith and prayer was a preferred method of coping for many and substituted the need for psychological help:

‘For me it was a lot of prayer, and being close to God, some people went to psychologists and psychiatrists, they sought psychological help, I didn’t’. (P10)

Advances in technology and research inspired optimism in one participant and others described a sense of hope when they met doctors and diabetes educators with T1DM. Other methods of support that the mothers would have valued included informative videos of other Saudi children with T1DM during a child’s hospitalization. A 24-hour support hotline to call in cases of hypo/hyperglycaemia, and face-to-face peer support in the few weeks after diagnosis would also have been beneficial.

#### 3.3.3 Transformation and adaptation

The transformational potential of “time” was described by many mothers as a key element in their adaptation. Time and adaptation were closely associated and involved a gradual process of adjustment from when knowledge was acquired, to when pain was eased, stress dissipated and a sense of normality and calm emerged. Many mothers reported these essential elements of their adaptation experience.

It was small everyday things that indicated adaptation. For example, one mother shared her 18 year old’s ability to drink coffee without sugar. Another was especially proud of eventually being able to speak of her experience without crying. The strength of participants’ experiences and their gradual recovery was eloquently summarized by one mother:

‘And there was never a case of a child diagnosed with T1DM where the parents were normal. They were all devastated, were emotionally destroyed and they all adapted and adjusted and became normal. Life doesn’t come to an end.’ (P8)

## 4. Discussion

This is the first qualitative study to explore the experiences of Saudi mothers who have a child with T1DM. Three key stages encompassed the mothers’ experiences: “being in the dark” characterized by emotional shock, the challenge of understanding T1DM, and attempts to rationalize its occurrence; “empowerment” during which they sought support and acquired new knowledge and skills; and “coping and acceptance” during which knowledge and skills were implemented, cultural perceptions and stigma were addressed, and there was movement towards a stage of adaptation.

The shock that parents experience when their child is diagnosed with T1DM and attempts to rationalize the diagnosis have been well documented (Iversen et al., 2018; Nordfeldt et al., 2013). A difference in this study was attribution of the “evil eye” as a causative factor and associated expressions of guilt because mothers had not taken precautions against it, which mirrors findings from a Tajkistan study by (Haugvik et al., 2017). A related point was the absence of terms such as grief or sadness when recalling a child’s diagnosis. In the KSA, adults have to appear stoic and have faith in God rather than express emotional vulnerability, suggesting these experiences are particular to the religious and social context of the Saudi culture.

Day-to-day management challenges were broadly similar to those reported in the wider literature. For example, the steep learning curve from the time of diagnosis (Iversen et al., 2018), overcoming the fear of injecting (Nordfeldt et al., 2013), fear of hypoglycaemia (Castensoe-Seidenfaden et al., 2017), sleep deprivation (Iversen et al., 2018), and children being judged and stigmatized (Rasmussen et al., 2008). A number of challenges were compounded by variable service provision including a lack of empathy from health care providers when receiving a child’s diagnosis, and inadequate or no training in the administration of injections. One mother had not been forewarned about parental sleep deprivation and not all children had access to school nurses. Superimposed on these difficulties is the mothers’ principle care giving role whilst fathers are the major decision-makers. Although one participant praised the support she had received from her husband this was not always the case and throws into sharp relief the distress and burden many mothers faced in the care of their child.

Participants felt they would have benefitted from different types of peer support that are not currently made available by services in the KSA. However, there is uncertainty regarding the most appropriate methods for delivering peer support. In a Swedish study, face-to-face meetings were preferred to remote internet connections, which respondents found to be impersonal (Nordfeldt et al., 2013), but not all the mothers in this study were in favour of face-to-face support. Uncertainties were expressed more generally in relation to psychological support, about which some participants were equivocal because of the importance of faith within Saudi culture. Some participants expressed a need for psychological support especially those who experienced the diagnosis of a second child with T1DM, while being close to God eliminated some mothers’ need for psychological support. In an Iranian study by (Abolhassani et al., 2013) also described how mothers coped by accepting God’s will, which emphasizes the need for alternative methods of emotional support to those provided in Western cultures. Similar findings were found by (Rossiter et al., 2019) in a study conducted in the United Arab Emirates (UAE) where mothers expressed how spiritual factors helped them cope after diagnosis of their child with T1DM. Moreover, it indicates the need for the assessment of diabetes related emotional distress in parents of children with T1DM and the provision of appropriate culturally appropriate support measures if necessary.

### 4.1. Study limitations

A limitation of this study was, given the context of the lead researcher’s background as a Saudi nurse, and as a peer re-living the experience during data collection, and whilst bracketing and reflexivity techniques were implemented throughout the study, setting her “epoche” meaning her judgements (Moustakas, 1994) aside, was difficult. Participants and the researcher may have shared a similar experience, under dissimilar circumstances with alternative perspectives (Polkinghorne, 2005). To attenuate this, the interview schedule was reviewed by the research team. Furthermore, throughout data analysis interpretations were discussed with the research team to attenuate bias.

The researcher-participant relationship is where the researcher is highly dependent on the participant ‘s willingness to share their knowledge about the research phenomena (Raheim et al., 2016). This interaction may place the researcher in a “superior” position and then to add the aspect of the venue being the lead researcher’s home to this dynamic can superimpose this hierarchy. This is another limitation in this study. In addition, to handle the shifts in power between research parties the practice of continuous reflexive awareness is paramount (Raheim et al., 2016) and this was considered throughout the interviews and throughout the study. Telephone interviews for data collection can limit the observation of nonverbal communication and inhibit further probing. However, this method supported the inclusion of women from diverse areas of the KSA and enhanced the range of experiences captured. An important caveat was the lack of participants from the private sector and non-urban settings. The transferability of the findings to these populations may therefore be limited.

### 4.2. Implications for practice

Better training for nurses, doctors, and allied health professionals to empathically communicate a T1DM diagnosis and equip parents with the skills and confidence to manage the condition, particularly the administration of injections, is necessary. More thorough discharge planning and consistent follow-up after initial diagnosis are also advised. Collaboration between the Ministry of Health and Ministry of Education is necessary to provide school nursing provision throughout the KSA and establish guidelines that ensure the safety of children with T1DM. Guidelines for the management and support of school aged children produced by the International Society for Paediatric and Adolescent Diabetes (Codner et al., 2018) are an important resource for this work.

A current gap in the KSA is a lack of platforms that facilitate peer support such as Diabetes UK. Hospitals could provide or signpost to such services at the time of diagnosis, particularly as families living with T1DM often know the most effective methods for managing the condition. In addition, there is a need to assess diabetes related distress especially for parents with two or more children with T1DM.

A study by (A. Al-Agha et al., 2011) successfully implemented a collaborative approach with frequent follow-up visits that helped achieve more optimal glycaemic control among children in the KSA. Thus, delivering family education of this type in addition to psychosocial support is needed as part of routine care.

## 5. Conclusion

Study findings have brought to light the experiences of Saudi mothers whose children have T1DM. Their accounts transcend geographical borders and highlight that irrespective of cultural differences, the experiences of mothers are broadly similar. Effective interventions delivered in other countries therefore stand a good chance of being effective in the KSA with specific adaptations for spiritual and cultural differences. The central role that Saudi mothers play in the management of a child’s condition indicates the need for customized methods of support that take into account the psychological and sociocultural needs of both mother and child. Further studies can build upon the current findings from this study. The focus of this study was mothers’ experiences however future studies can build upon this and perhaps explore fathers’ experiences. In addition, future studies can take this further and assess and explore the impact of diabetes on the psychosocial aspects of diabetes care for parents of children with T1DM within the Saudi cultural context. Furthermore, there is a possibility that some of the findings from this study could translate to mothers of a similar cultural and religious background in other geographical areas.
